# Multi-delay ASL can identify leptomeningeal collateral perfusion in endovascular therapy of ischemic stroke

**DOI:** 10.18632/oncotarget.13898

**Published:** 2016-12-10

**Authors:** Xin Lou, Songlin Yu, Fabien Scalzo, Sidney Starkman, Latisha K. Ali, Doojin Kim, Neal M. Rao, Jason D. Hinman, Paul M. Vespa, Reza Jahan, Satoshi Tateshima, Nestor R. Gonzalez, Gary R. Duckwiler, Jeffrey L. Saver, Bryan Yoo, Noriko Salamon, Jinhao Lyu, Lin Ma, Danny JJ Wang, David S Liebeskind

**Affiliations:** ^1^ Department of Radiology, Chinese PLA General Hospital, Beijing, China; ^2^ Department of Neurology, UCLA Stroke Center, Los Angeles, California, USA; ^3^ Department of Radiology, UCLA Stroke Center, Los Angeles, California, USA; ^4^ Department of Medical Center, UCLA Stroke Center, Los Angeles, California, USA; ^5^ Department of Neurosurgery/Interventional Neuroradiology, UCLA Stroke Center, Los Angeles, California, USA; ^6^ Department of Radiology and Neurosurgery, UCLA Stroke Center, Los Angeles, California, USA

**Keywords:** collateral circulation, stroke, perfusion imaging, arterial spin-labeling, cerebral blood flow

## Abstract

**Background and Purpose:**

Multi-delay arterial spin-labeling (ASL) perfusion imaging has been used as a promising modality to evaluate cerebral perfusion. Our aim was to assess the association of leptomeningeal collateral perfusion scores based on ASL parameters with outcome of endovascular treatment in patients with acute ischemic stroke (AIS) in the middle cerebral artery (MCA) territory.

**Materials and Methods:**

ASL data at 4 post-labeling delay (PLD) times (PLD = 1.5, 2, 2.5, 3 s) were acquired during routine clinical magnetic resonance examination on AIS patients prior to endovascular treatment. A 3-point scale of leptomeningeal collateral perfusion grade on 10 anatomic regions was determined based on arterial transit times (ATT), cerebral blood flow (CBF), and arterial cerebral blood volume (CBV), estimated by the multi-delay ASL protocol. Based on a 90-day modified Rankin Scale (mRS), the patients were dichotomized to moderate/good (mRS 03) and poor outcome (mRS 46) and the regional collateral flow scores were compared.

**Results:**

Fifty-five AIS patients with unilateral MCA stroke (mean 73.9514.82 years) including 23 males were enrolled. Compared with poor outcome patients, patients with moderate to good outcomes had a significantly higher leptomeningeal collateral perfusion scores on CBV (3.012.11 vs. 1.821.51, p=0.024) but no differences on scores on CBF (2.311.61 vs. 1.661.32, p=0.231) and ATT (2.672.33 vs. 3.423.37, p=0.593).

**Conclusions:**

Higher leptomeningeal collateral perfusion scores on CBV images by ASL may be a specific marker of clinical outcome after endovascular treatment in patients with acute MCA ischemic stroke. Further study with larger sample size is warranted.

## INTRODUCTION

In acute ischemic stroke, the collateral circulation alleviates ischemic injury to tissue and may keep the tissue viable during a vulnerable but potentially salvageable state (penumbra) [1]. Leptomeningeal collaterals are anastomotic vessels providing alternative routes and play an important role in maintaining the cerebral circulation during acute stroke and chronic hypoperfusion [2]. In acute stroke, the presence of leptomeningeal collaterals is associated with better outcomes after thrombolysis [3]. There are several existing studies using a number of imaging modalities and grading methods, to evaluate leptomeningeal collaterals in acute stroke [4]. Conventional angiography remains the reference standard to measure collateral extent and vessel number, with alternative CT, MRI and transcranial Doppler (TCD) based techniques, yet there is no accepted standard or routine imaging technique to quantify the extent of collateral circulation [5].

Arterial spin-labeling (ASL) is a noninvasive technique that can quantify regional cerebral blood flow (CBF) with high reproducibility. The bright intravascular signal known as arterial transit artifact (ATA) on ASL may identify the presence and degree of collateral perfusion in Moyamoya disease [6]. As a recently developed ASL technique, background suppressed three-dimensional pseudo-continuous arterial spin-labeling (3D pCASL) offers improved signal-to-noise ratio (SNR) and reduced susceptibility to transit time variability compared with standard 2D pCASL [7].

Several recent studies have evaluated the clinical utility of 3D pCASL with multiple post-labeling delays (PLD) in acute ischemic stroke [8–10]. Compared with ASL with single delay, 3D pCASL with multiple PLDs may provide improved visualization of collateral flow in acute stroke via adynamic image series and the potential for quantitative assessment of collateral perfusion using parameters of CBF, cerebral blood volume (CBV), and arterial transit time (ATT). To date, however, there is no study to evaluatethe clinical utility of leptomeningeal collateral scores using multi-delay 3D pCASL in acute stroke. Therefore, our primary aim was to assess the association of leptomeningeal collateral perfusion scores based on ASL parameters including CBF, CBV, and ATT with clinical outcomes of thrombolysis/endovascular treatment in patients with acute ischemic stroke.

## MATERIALS AND METHODS

### Patients

The present analysis was performed on data collected from February 2012 to August 2013 in an ongoing prospective registry of patients evaluated with MRI at a comprehensive stroke center. The study was approved by the local Institutional Review Board. Image data were included in this study if: (1) the patient was diagnosed as acute middle cerebral artery (MCA) stroke; (2) baseline MRI was performed within 24 hours of symptom onset; (3) the multi-delay multi-parametric 3D pCASL perfusion imaging was acquired along with routine clinical MRI, and was performed prior to thrombolysis or endovascular treatment; (4) the absence of previous intracranial hemorrhage, brain surgery, or large territorial lesion.

### MRI protocol

All patients underwent MRI on Siemens 1.5 T Avanto or 3.0 T TIM Trio systems (Erlangen, Germany), using 12 channel head coils. The MRI protocol included diffusion weighted imaging (DWI), T2 weighted imaging (T2WI), fluid attenuated inversion recovery (FLAIR), and perfusion-weighted imaging sequences. ASL scans were performed using a 4-delay pCASL protocol with background suppressed 3D GRASE (gradient and spin echo) readout (labeling pulse duration = 1.5 s, PLD = 1.5/2/2.5/3 s, TR = 3.5/4/4.5/5 s, FOV = 22 cm, matrix = 64 × 64, 26 × 5mm slices, rate-2 GRAPPA, TE = 22 ms, 8 pairs of tag and control for each delay, total scan time 4 min 30 s). Two global inversion pulses were applied during the PLD for background suppression. The first background suppression pulse was applied immediately after the labeling pulses, while the interval of the second background suppression pulse was adjusted based on the PLD, T1 of gray and white matter to achieve ~85% suppression for gray- and white-matter signals [9].

### Imaging review

**Figure 1:**
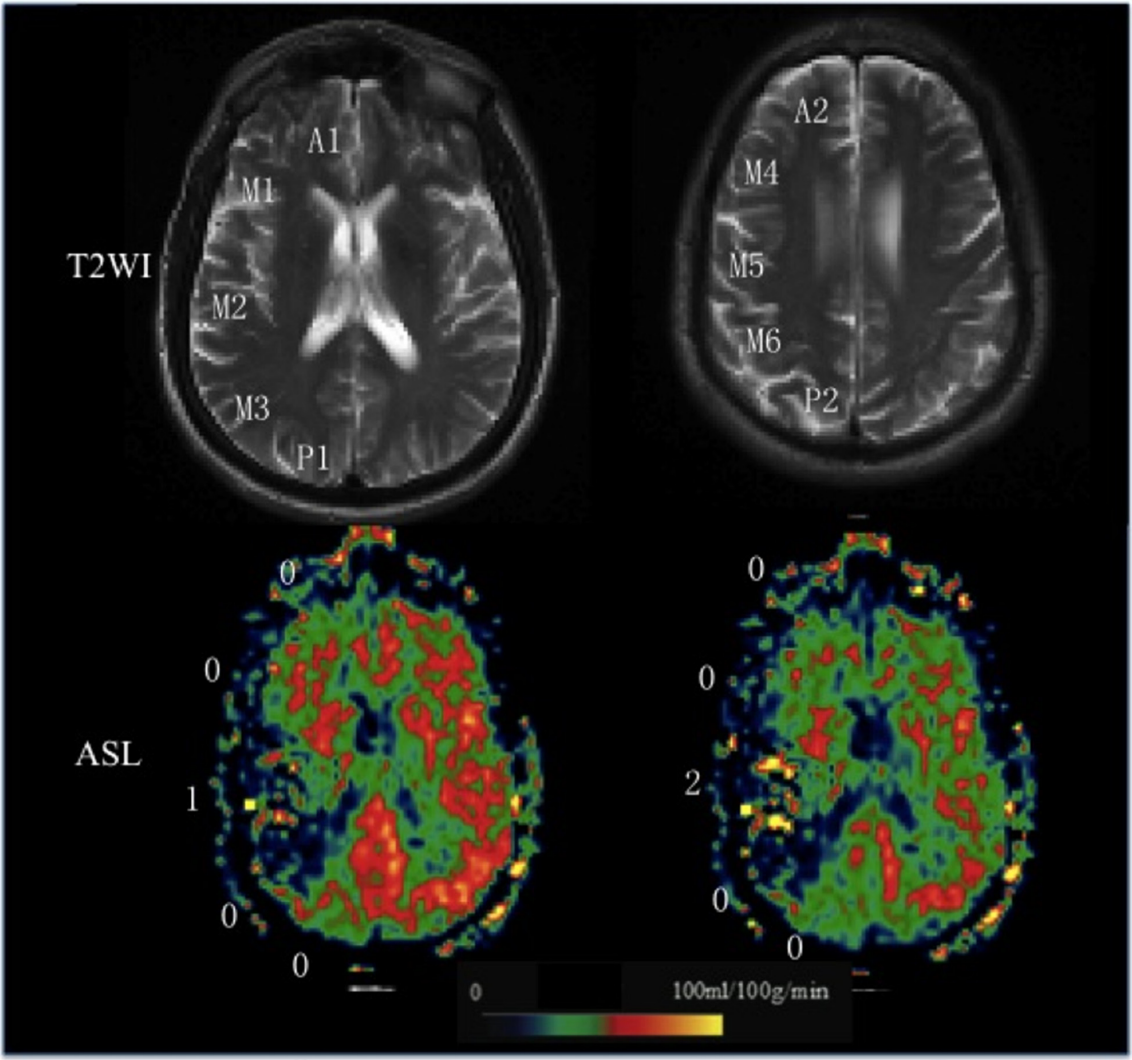
Grading scale illustrations A 65-year-old man with right MCA stroke. T2WI shows 2 ASPECTS levels are used in the current study. ASL collateral consensus scores are shown as an illustration of the grading scale (0, no or minimal arterial transit artifact (ATA) signal; 1, moderate ATA; 2, Robust ATA).

The CBF, ATT, and CBV maps of ASL were post-processed offline using custom software programs that was performed with Interactive Data Language (IDL (Boulder, CO, USA)) software programs developed in-house. Two slices corresponding to Alberta Stroke Program Early Computed Tomography Score (ASPECTS) were selected [6]. Ten regions of interest (ROI) of M1-6, A1-2, and P1-2 depiction were performed using the free software ImageJ (V1.37, National Institutes of Health, USA). We chose to focus only on cortical regions because of the difficulties in visualizing ASL signals in the white matter and basal ganglia region. Each ROI was drawn manually on the lesion side by referring to a previous study [6]. The same ROIs at each side were applied to the CBF, ATT, and CBV maps. Based on these images, a 3-point scale of 10 territories was visually categorized as follow: 0: no or minimal ATA signal; 1: moderate ATA signal; 2: robust ATA signal (Figure 1). ATA was defined as linear or serpentine hyperintense signal in the cortical region.

Two neuroradiologists (X.L. and S.Y.) who were blinded to clinical findings separately reviewed all MR images for leptomeningeal collateral perfusion scores on CBV, CBF, and ATT. The baselines features and 90 days mRS scores were collected.

### Statistical analysis

Student's t-test or the Mann-Whitney U-test (when continuous variables had skewed distributions) was used to identify the difference in the continuous variables. The difference in each of the categorical variables between the two groups was tested with χ² or Fisher's exact tests (when the expected cell frequency was < 5). A two-tailed P value less than 0.05 was considered statistically significant. All analyses were performed using the software SAS 9.2 (SAS Institute Inc., Cary, NC, USA).

## RESULTS

From February 2012 to August 2013, 55 eligible patients were enrolled (age 73.95±14.82 yr., 23 male). Among them, 53 patients had an available demographic, clinical, and laboratory information. Hypertension is the most common risk factor (33/53, 62.3%). For all 55 patients, the time form the last known well time to treatment was187±171.3 minutes. The types of treatment included IV tissue plasminogen activator (tPA) in 43 patients (78.2%), IA tPA in 4 patients (7.2%), retrieval device in 19 patients (34.5%), and angioplasty and stent in 3 patients (5.5%). In 90 days follow-up, 23 patients had a mRS 0-3 and 32 patients had a mRS 4-6 (see Supplemental Table I for more details).

**Figure 2:**
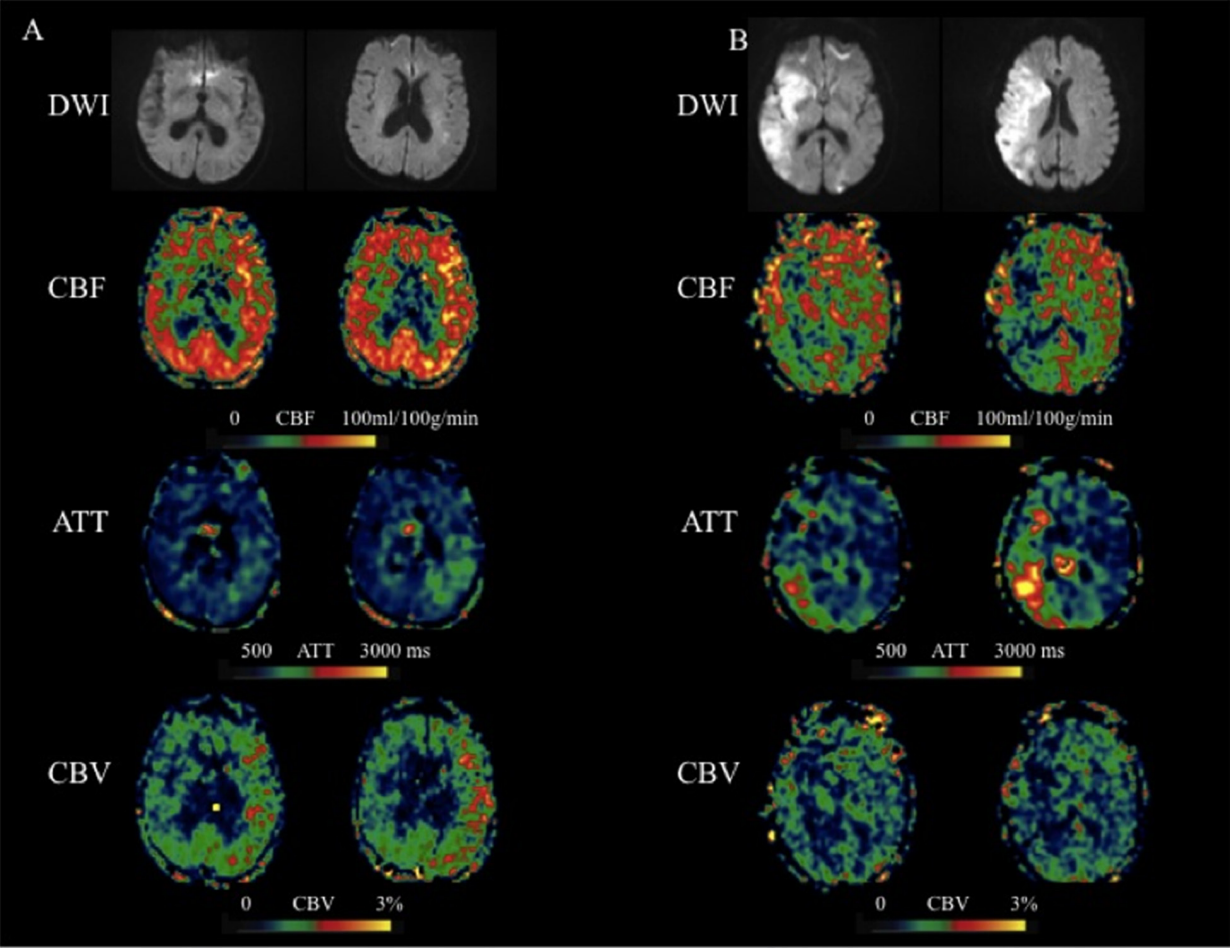
Two representative cases with acute MCA stroke **A.** An 83-year-old female presented with weakness of right side and was found to have a left MCA stroke with a 90 days mRS of 2. IV tPA was given 3 hours after onset. ASL scanned 4.6 hours after onset showed hyper-perfusion in the left MCA region. Based on the CBF, ATT, and CBV maps, the scores of 6 left MCA territories are 9, 4 and 12 respectively. **B.** An 85-year-old female presented with aphasia, hemianopsia, weakness of left side and was found to have a right MCA stroke with a 90 days mRS of 6. IV tPA was given 3 hours after onset. ASL scanned 4.7 hours after onset showed hypo-perfusion in the right MCA region. Based on the CBF, ATT, and CBV maps, the scores of 6 right MCA territories are 4, 6 and 1 respectively.

The sum of the ten regions score in each patient was calculated to represent the collaterals on CBF, ATT and CBV respectively. There was good agreement between the 2 readers (к = 0.83-0.92) for each parameter. Compared with those with poor outcome, patients with moderate to good outcomes had a significantly higher leptomeningeal collateral flow scores on CBV (3.01±2.11 vs. 1.82±1.51, p = 0.024) but no differences on scores on CBF (2.31±1.61 vs. 1.66±1.32, p = 0.231) and ATT (2.67±2.33 vs. 3.42±3.37, p = 0.593) (Figure 2).

Among features of risk factors, medication, and type of treatment, the patients with good outcome had a younger age (69.34±14.92 yr. vs. 77.27±14.05, p = 0.049) compared with those with poor outcome, but no significant difference in the other features (Table 1).

**Table 1:** Comparison baseline characteristics, treatment features, and collateral perfusion scores between patients with good outcome and poor outcome

Characteristic	Patients with good outcome (N=23)	Patients with bad outcome (N=32)	*p*
Age— yr	69.34±14.92	77.27±14.05	0.049
Male sex — no. (%)	7(30.4%)	16(50.0%)	0.147
Risk factors— no. (%)*			
Hypertension	12/23(52.2%)	21/30(70.0%)	0.185
Hyperlipidemia	7/23(30.4%)	11/30(36.7%)	0.635
Diabetes mellitus	4/23(17.4%)	10/30(33.3%)	0.192
Smoking history			
Current	3/23(13.0%)	4/30(13.3%)	1.000
Former	5/23(21.7%)	4/30(13.3%)	0.661
Never	15/23(65.2%)	22/30(73.3%)	0.524
Atrial fibrillation	8/23(34.8%)	10/30(33.3%)	0.912
Stroke history	3/23(13.0%)	8/30(26.7%)	0.384
Blood pressure — mm Hg*			
Systolic	156.7±30.20	151.2±24.53	0.712
Diastolic	78.3±16.92	86.5±17.23	0.138
Medication— no. (%)*			
Aspirin	11/23(47.8%)	14/30(46.7%)	0.933
Warfarin	4/23(17.4%)	0/30(0.0%)	0.064
Qualifying artery — no.			
LMCA	15	20	0.836
RMCA	8	12	0.836
Time from onset to treatment— min	209±177.6	187±154.0	0.654
Type of treament — no. (%)			
IV tPA	18(78.3%)	25(78.1%)	0.990
IA tPA	2(8.7%)	2(6.2%)	1.000
Retrieval device	8(34.8%)	11(34.4%)	0.910
Angioplasty/stent	2(6.3%)	1(4.3%)	0.768
DWI lesion volume — ml†	8.89±8.64	36.39±47.62	0.060
NIHSS‡	16(6-20)	19(12-24)	0.191
Leptomeningeal collateral flow scores			
CBV	3.01±2.11	1.82±1.51	0.024
CBF	2.31±1.61	1.66±1.52	0.231
ATT	2.67±2.33	3.42±3.37	0.593

Plus–minus values are means ±SD. *The data came from 53 patients with available risk factors, blood pressure, and medication. †The data came from 44 patients with available DWI. ‡The data came from 45 patients with available NIHSS. tPA denotes tissue plasminogen activator; CBV denotes cerebral blood volume; CBF denotes cerebral blood flow; ATT denotes arterial transit times.

## DISCUSSION

Our study provides the initial description of collateral perfusion graded by quantitative leptomeningeal collateral scores on multiple parameters including CBF, ATT, and CBV using ASL with multiple PLDs in patients with acute MCA stroke. This study showed that higher leptomeningeal collateral perfusion score on CBV by using multi-delay ASL is associated with better outcomes (mRS 0-3). CBV is a hemodynamic parameter describing the interaction effect between CBF and ATT, and it reflects the arterial blood volume from the labeling plane to the imaging pixel. Several studies have shown that the CBV map depicts the lesions visualized on DWI, helping predict potentially infarcted brain tissue that is not salvageable despite reperfusion [11]. The CBV using this 4-delay pCASL protocol may also be more sensitive than CBF for characterizing chronic stroke and brain tumor [12]. In this study, however, a receiver operator curve analysis was not used to determine a cut-off values of leptomeningeal collateral perfusion score on CBV to predict outcome because of a limited sample size.

Our study revealed that patients with better outcome have more robust leptomeningeal collateral perfusion scores on CBF and lower scores on ATT when compared with patients with worse outcome, although the differences were not significant. In this study, the estimated CBF is the mean of ATT adjusted CBF at 4 PLDs, there should be a minimal penalty in SNR compared to pCASL scans performed at a single PLD, assuming the same amount of scan time. ATT is quantified through a weighted delay approach [8], providing access to a novel physiological parameter that likely reflects the recruitment of collateral flow sources. Based on the definition, CBV is the product of CBF and ATT. Therefore, CBV map may enhance the ATA if it is present in both CBF and ATT maps (Figure 2A). On the other hand, if the ATA is only present in CBF or ATT map (or spatially not overlap), then the ATA effect may cancel or be reduced on CBV maps (Figure 2B), suggesting the patient's arteries are not dilated. Overall, the findings from the present study suggest that the collateral scores based on CBV maps are more sensitive and potentially more reliable than those on CBF or ATT maps for predicting the clinical outcome at 90-day follow-up. CBV is also a routine parameter of conventional perfusion imaing (perfusion weighted imaging and CT perfusion), it is appealing to test whether CBV derived from the conventional perfusion imaing is as predictive as CBV derived from multi-PLD ASL.

A good agreement between the inter-observer reliability for each parameter was demonstrated in our study. So far, there is only one study of ASL-based grading methods to assess the leptomeningeal collateral flows for acute stroke patients, which has not been replicated nor has inter-observer reliability been graded, and it remains to be seen if they represent robust means of assessing collateral flow [12]. In this study, the 10 regions based on ASPECTS were selected to calculate the total leptomeningeal collateral perfusion score. The rationale for this approach is based on the fact that in patients with MCA acute stroke, the pial or leptomeningeal branches from anterior and posterior cerebral arteries to the MCA territory play a pivotal role.

We noted that patients with good outcome were younger compared with those with poor outcome. It is obvious that advancing age has a major negative impact on stroke morbidity, mortality, and long-term outcome [13]. Ongoing studies are investigating other correlations with the human collateral circulation of the brain across a variety of stroke populations [14].

Several limitations should be acknowledged, including the limited sample size which might not be power enough to do the multivarible analysis and consequent caution that preclude definitive conclusions. Second, the choice of the precise time parameters including PLD and delayed filling time was also empirical, as no standards exist. However, a reliable perfusion cannot be estimated accurately using the present pCASL protocol if ATT is greater than 3 s. Third, our study may not express all regions supplied by the leptomeningeal collaterals, only two representative slices were selected for quantitative measures, Finally, we had not investigated the consistency of CBV score and conventional angiography collateral grading or other collateral grading approaches, further studies are expected.

## CONCLUSIONS

In brief, higher leptomeningeal collateral perfusion scores on CBV images by ASL may be associated with good outcome of thrombolysis/endovascular treatment in patients with acute MCA ischemic stroke. Prospective studies are indicated to investigate whether this parameter can effectively stratify therapeutic algorithms for those individuals with bad outcome.

## Abbreviations

PLD: post-labeling delay times
3D pCASL: three-dimensional pseudo-continuous arterial spin-labeling
ATT: arterial transit time
ATA: arterial transit artifact.
